# Ciliogenesis and cerebrospinal fluid flow in the developing *Xenopus* brain are regulated by *foxj1*

**DOI:** 10.1186/2046-2530-2-12

**Published:** 2013-09-24

**Authors:** Cathrin Hagenlocher, Peter Walentek, Christina M?ller, Thomas Thumberger, Kerstin Feistel

**Affiliations:** 1 Institute of Zoology, University of Hohenheim, Garbenstr. 30, Stuttgart 70593, Germany; 2 Present address: Department of Molecular and Cell Biology, Center for Integrative Genomics, University of California at Berkeley, Berkeley, California 94720, USA; 3 Centre for Organismal Studies, Im Neuenheimer Feld 230, University of Heidelberg, 69120 Heidelberg, Germany

**Keywords:** Cilia, Brain, Cerebrospinal fluid flow, Choroid plexus, *Foxj1*, Hydrocephalus, Reissner?s fiber, Subcommissural organ, *Xenopus*, Zona limitans intrathalamica

## Abstract

**Background:**

Circulation of cerebrospinal fluid (CSF) through the ventricular system is driven by motile cilia on ependymal cells of the brain. Disturbed ciliary motility induces the formation of hydrocephalus, a pathological accumulation of CSF resulting in ventricle dilatation and increased intracranial pressure. The mechanism by which loss of motile cilia causes hydrocephalus has not been elucidated. The aim of this study was: (1) to provide a detailed account of the development of ciliation in the brain of the African clawed frog *Xenopus laevis*; and (2) to analyze the relevance of ependymal cilia motility for CSF circulation and brain ventricle morphogenesis in *Xenopus*.

**Methods:**

Gene expression analysis of *foxj1*, the *bona fide* marker for motile cilia, was used to identify potentially ciliated regions in the developing central nervous system (CNS) of the tadpole. Scanning electron microscopy (SEM) was used to reveal the distribution of mono- and multiciliated cells during successive stages of brain morphogenesis, which was functionally assessed by bead injection and video microscopy of ventricular CSF flow. An antisense morpholino oligonucleotide (MO)-mediated gene knock-down that targeted *foxj1* in the CNS was applied to assess the role of motile cilia in the ventricles.

**Results:**

RNA transcripts of *foxj1* in the CNS were found from neurula stages onwards. Following neural tube closure, *foxj1* expression was seen in distinct ventricular regions such as the zona limitans intrathalamica (ZLI), subcommissural organ (SCO), floor plate, choroid plexus (CP), and rhombomere boundaries. In all areas, expression of *foxj1* preceded the outgrowth of monocilia and the subsequent switch to multiciliated ependymal cells. Cilia were absent in *foxj1* morphants, causing impaired CSF flow and fourth ventricle hydrocephalus in tadpole-stage embryos.

**Conclusions:**

Motile ependymal cilia are important organelles in the *Xenopus* CNS, as they are essential for the circulation of CSF and maintenance of homeostatic fluid pressure. The *Xenopus* CNS ventricles might serve as a novel model system for the analysis of human ciliary genes whose deficiency cause hydrocephalus.

## Background

Cerebrospinal fluid (CSF) is a clear liquid characterized by high ion and protein content that fills the ventricular system and subarachnoid space of the brain. Following neural tube closure, enlargement of the ventricles is driven by CSF secretion from ependymal cells. During later development, CSF is primarily secreted by the specialized epithelia of the highly vascularized choroid plexus (CP). CSF provides mechanical buffering to the brain, transports signaling molecules, and eliminates waste products from the ependyma [1]. For this purpose, CSF generated inside the ventricles has to be actively transported through the system of lateral, third, and fourth ventricles into the subarachnoid space, where it is reabsorbed into the venous system [2].

In patients suffering from hydrocephalus this process is disturbed, resulting in increased intra-ventricular pressure, enlargement of the lumina, and damage to brain tissue. Hydrocephalus may result from an overproduction of CSF, impaired reabsorption or by obstruction of any of the ventricular ducts and foramina, that is, the narrow passageways through which CSF flows from one ventricle to the next as well as into the subarachnoid space [3]. Obstructions may be caused by hemorrhage, tumors, or morphogenetic malformations of ventricle-contacting tissues [3]. Immotile cilia result in impaired CSF transport (ependymal flow) as well, as encountered in certain ciliopathies [4,5]. Ciliary immotility leading to hydrocephalus has been shown to result from mutations in structural proteins, such as Spag6 [6] or axonemal motor proteins such as Dnahc5. Mice deficient in *Mdnah5*[7] for example have no detectable ependymal flow, ultimately leading to stenosis of the cerebral aqueduct and hydrocephalus in the lateral and third ventricles [5,7]. On the regulatory level, transcription factors of the regulatory factor X (RFX) and winged-helix (forkhead, FOX) families govern the differentiation of cells towards a ciliated phenotype. RFX3-deficiency leads to aberrant ciliation and hydrocephalus in mice [8]. The winged-helix family member *foxj1* acts as a master regulator of genes inducing the biogenesis of motile cilia [9]. In zebrafish and mouse, loss of *Foxj1* function leads to a loss of motile, but not immotile cilia [9-12], and *Foxj1* knock-out mice develop hydrocephalus postnatally [10,11]. Transcription of *Foxj1* marks the onset of ciliogenesis in all embryonic tissues studied so far, rendering *Foxj1* a *bona fide* marker gene for motile cilia [9].

The present study provides a detailed account of the development of motile cilia in the *Xenopus* tadpole brain, up to the onset of metamorphosis. Cilia-driven CSF flow was assessed by video microscopy following injection of fluorescent microspheres into the ventricles. CSF flow was compromised in *foxj1* morphants, which developed hydrocephalus at tadpole stage. Our study provides an entry point into using *Xenopus* as a model system for studying human ciliary genes whose deficiency cause hydrocephalus.

## Methods

### Animals

All animals were treated according to the German regulations and laws for care and handling of research animals, and experimental manipulations according to ? 6, article 1, sentence 2, No. 4 of the Animal Protection Act were approved by the Regional Government Stuttgart, Germany (Vorhaben A 365/10 ZO ?Molekulare Embryologie?).

### RNA *in situ* hybridization and histological analysis

Embryos were fixed for 2 h in 1 ? MEMFA consisting of one part of 10 ? MEMFA (1 M MOPS, pH 7.4, Roth; 20 mM EGTA; 10 mM MgSO_4_, both Applichem), one part formaldehyde (37%, Roth) and eight parts H_2_O. Where indicated, larval brains were explanted and embryos as well as explants were processed following standard protocols [13]. A digoxigenin (Roche)-labeled RNA probe was prepared from linearized plasmid containing the full-length sequence of *foxj1*[14] using T7 RNA polymerase (Promega). *In situ* hybridization was performed according to [15]. For histological analysis embryos were embedded in gelatin-albumin and sectioned on a vibratome at 30 to 40 ?m.

### SEM analysis

For scanning electron microscope (SEM) analyses, brains were explanted, bisected, further processed as described [16] and viewed at 10 kV on a Jeol SEM.

### MO-mediated knock-down of *foxj1*

One pmol of *foxj1* antisense morpholino oligonucleotide (MO, Gene Tools, Philomath, OR, USA) was injected into the animal region of each dorsal blastomere of four to eight cell-stage embryos using a Harvard Apparatus setup. The *foxj1*MO targeted the initiation codon as described in [14]. Dextran tetramethylrhodamine (MW 70.000, 0.5-1 ?g/?L, Invitrogen) was used as a lineage tracer. In all experiments, care was taken to exclusively use embryos with a clear dorsoventral segregation of pigment [17], and only correctly targeted specimens (controlled by lineage tracer fluorescence in the CNS at stage 21) were processed for further analysis.

### Measurement of ventricles

Width and length of the fourth ventricle were measured in ImageJ (http://imagej.nih.gov/ij/) [18].

### CSF flow analysis

Embryos were anesthetized using benzocaine (E1501, Sigma). Fluorescent microbeads (F8823, FluoSpheres? carboxylate-modified microspheres, 1.0 ?m, yellow-green fluorescence (505/515), 2% solids, Invitrogen, Molecular Probes) were diluted 1/250 into a solution of 0.5-1 ?g/?L dextran tetramethylrhodamine in 1 ? MBSH. A volume of about 20 nL was injected into the fourth ventricle using a pulled-out glass micropipette. Glass pipettes (Hilgenberg) were prepared using a Sutter Instruments Flaming/Brown micropipette puller (P-87; heat 720, pull 30, velocity 290, time 3) to yield pipettes with a short taper and short tip for enhanced stability. Embryos were embedded in 1 ? MBSH buffer. The coverslip was adjusted in height using vaseline as a flexible spacer. Movement of beads was documented on a Zeiss Axioskop 2 (Zeiss) using a 20 ? Plan-Neofluar objective (Zeiss) by taking 1,000 frame-long time-lapse movies at 175 fps using a Zeiss Axiocam HSm (size of region of interest, ROI, 660 ? 128 px) and Axiovision 4.7 together with the ?fast recorder? plug-in.

To visualize the directionality of moving beads, 20 consecutive raw frames were color-coded from red to green (green representing the current position of the particle) and projected onto a single frame by using a custom interpretation of the ?Temporal-Color Coder? (Kota Miura, EMBL, Heidelberg) plug-in for Fiji [19]. Particle movement was analyzed towards velocity of single beads using the ImageJ plug-in ParticleTracker [18,20], which saves the coordinates of the trajectories. All trajectories matching the analysis criteria were then further processed with the help of a custom-made program written in statistical R (The R project for statistical computing; http://www.r-project.org/) which calculated the length and velocity of each particle track [13].

### Statistical analysis

*P* values were calculated using the Mann?Whitney U-test in statistical R. In order to account for multiple testing, *P* values are reported after strict bonferroni correction at nominal levels * <0.05, ** <0.01, and *** <0.001 (adjusted levels * <0.0166, ** <0.0033, *** <0.00033 for *n* = 3).

## Results

### *Xenopus* tadpoles express *foxj1* in distinct regions of the developing CNS

To establish an overview of the sites where motile cilia reside in the developing *Xenopus* brain, we analyzed *foxj1* mRNA expression using whole-mount *in situ* hybridization. Neural expression was first seen shortly after gastrulation, when transcripts were detectable in the midline of the neural plate (Figure?1A; see also [21]). Midline expression persisted in stage 20 embryos, when the neural tube has closed, in the floor plate of the deuterencephalon, that is, the part of the CNS which develops in close contact to the underlying notochord (Figure?1B, C). Transcripts were absent from the archencephalon, which will give rise to the forebrain and is situated dorsally to the prechordal plate at stage 20 (Figure?1B, C). *foxj1* signals persisted in the floor plate of the spinal cord throughout embryonic development (Figure?1D-G, outlined arrowheads in D?-F?). A considerably stronger expression was always apparent in the caudal-most part of the spinal cord (arrowheads in Figure?1D-F), corresponding to the ampulla terminalis, a ciliated vesicular widening of the spinal cord central canal [22].

**Figure 1:**
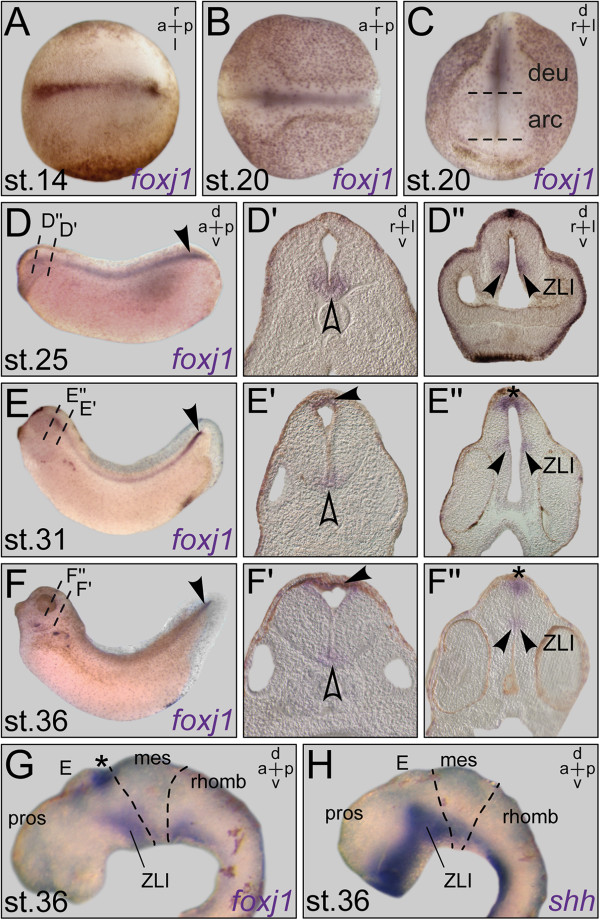
***Xenopus *tadpoles express *foxj1 *in distinct regions of the developing CNS.** Whole-mount *in situ* hybridization of staged embryos and explanted brains. **(A)** Expression at early neurula stage (dorsal (d) view). **(B, C)** Expression at late neurula stage in epidermis and floor plate, in dorsal **(B)** and frontal view **(C)**. **(D)** Staining in the floor plate at stage 25. Note the higher expression level at the ampulla terminalis (arrowhead). **(D?, D?)** Transversal sections, levels indicated in **(D)**, revealed staining in the ventral (v) midline (outlined arrowhead) **(D?)** and the zona limitans intrathalamica (ZLI; arrowheads) **(D?)**. **(E, F)** Expression at stage 31 **(E)** and stage 36 **(F)** in the nephrostomes, the spinal cord, and the ampulla terminalis (arrowheads). **(E?, E?, F?, F?)** Histological sections as indicated in **(E, F)** highlighted expression in the rhombencephalic roof (arrowheads in **E?, F?**), the ventral midline (outlined arrowheads in **E?, F?**) as well as in the subcommissural organ (SCO; asterisk in **E?, F?, G)** and the ZLI (arrowheads in **E?, F?)**. **(G, H)** Brain explants at stage 36 after *in situ* hybridization with antisense probes for *foxj1***(G)** and *shh***(H)** in side view showed co-localization of mRNA expression in the ventral midline and the ZLI. a = anterior; arc = archencephalon; deu = deuterencephalon; l = left; mes = mesencephalon; p = posterior; pros = prosencephalon; rhomb = rhombencephalon; r = right; st. = stage.

During cephalic ventricle inflation and flexion (stage 25), a bilateral *foxj1* domain was seen in the minor groove of the cephalic flexure (arrowhead in Figure?1D?), which represents the rostral continuation of the spinal cord and rhombencephalic floor plate. This prosencephalic expression persisted through stage 36 (arrowheads in Figure?1E?, F?). When stage 36 brain explants were probed for *foxj1* (Figure?1G) or *shh* (Figure?1H) expression, respectively, domains were found to overlap, identifying the *foxj1*-positive area in the future diencephalon as the zona limitans intrathalamica (ZLI; [23]). The ZLI represents an important signaling center for diencephalon development [24].

A second prominent expression domain of *foxj1* was found in the subcommissural organ (SCO), located dorsally at the boundary between di- and mesencephalon, that is, the entrance of the cerebral aqueduct [25]. Expression in the SCO started around stage 31 (Figure?1E, asterisk in E?) and persisted throughout pre-metamorphosis, visible as a round patch of strong staining in dorsal views (asterisk in Figure?1F?, G; arrowheads in Figure?2A, Additional file 1: Figure S1, Figure?3A, Figure?4A). At about the same time (stage 31), *foxj1* signals were first detected in the ependymal cell layer located at the rhombencephalon roof (arrowhead in Figure?1E?). This expression gradually intensified (arrowhead in Figure?1F?) and was still detectable in the single-layered ependyma of stage 53 embryos underneath the well-developed tissue of the fourth ventricle choroid plexus (CP; Figure?3A??, A??, Figure?4A??, A??).

**Figure 2:**
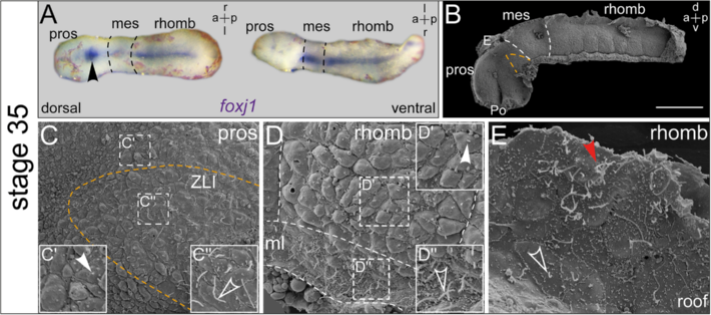
***foxj1 *expression correlates with elongation of monocilia and the emergence of multiple cilia. ***In situ* hybridization and scanning electron microscopy (SEM) on explanted brains at stage 35. **(A)** Explant shown in dorsal (d) and ventral (v) view. Strong expression in the ventral midline and the subcommissural organ (SCO; arrowhead). **(B)** SEM picture of brain explant dissected sagittally with view onto the ventricular surface, the zona limitans intrathalamica (ZLI) is delimited with orange dashed line and boundaries between brain regions are indicated by white dashed lines. Bar represents 200 ?m. **(C)** Overview and enlargements show short primary cilia on cells in the prosencephalic region (arrowhead in **(C?)**), and elongated monocilia on cells within the ZLI (outlined arrowhead in **(C?)**). **(D)** Close-up view onto the ventral aspect of a single hindbrain rhombomere with indicated boundaries and midline (ml). Enlargements showing short cilia on the rhombomere (arrowhead in **(D?)**) as well as elongated cilia in the ventral ml (outlined arrowhead in **(D?)**). **(E)** Close-up view onto the rhombencephalon (rhomb) roof with elongated monocilia (outlined arrowhead) and first MCCs (arrowhead). a = anterior; E = epiphysis; l = left; mes = mesencephalon; p = posterior; Po = preoptic region; pros = prosencephalon; r = right.

**Figure 3:**
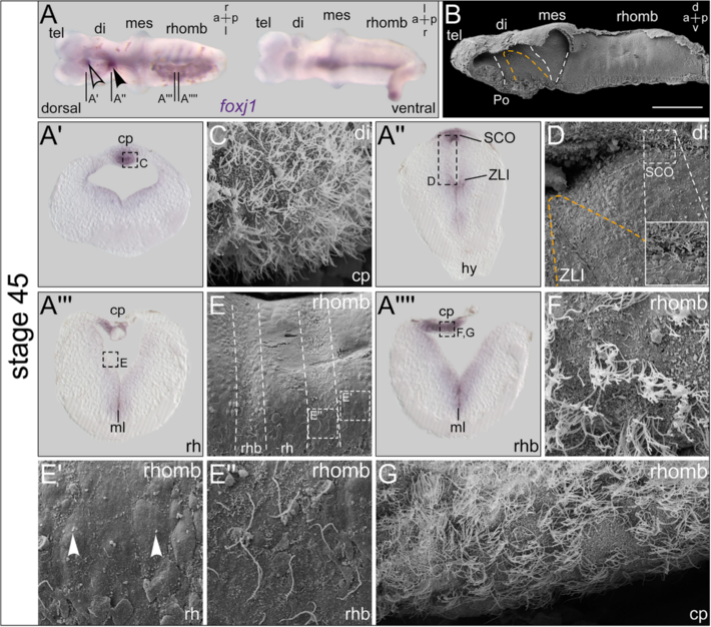
**Additional *foxj1 *expression domains identify regions with emerging ciliation. ***In situ* hybridization and scanning electron microscopy (SEM) on explanted brains at stage 45. **(A)** Explant shown in dorsal (d) and ventral (v) view. Expression in the ventral midline, rhombomere boundaries, subcommissural organ (SCO, arrowhead), and the choroid plexus (cp; outlined arrowhead). **(B)** SEM picture of brain explant dissected sagittally with view onto the ventricular surface, the zona limitans intrathalamica (ZLI) is delimited with orange dashed line and boundaries between brain regions are indicated by white dashed lines. Bar represents 200 ?m. **(A?-A??)** Transversal histological sections as indicated in **(A)**, dorsal side up, and SEM pictures **(C-G)** of corresponding regions as indicated in the sections, respectively. **(C)** MCCs on cp invaginating from the diencephalon roof. **(D)** Close-up view onto the ZLI region; enlargement showing ciliated structure of the SCO. **(E)** Close-up view revealing metamerical organization of the rhombencephalon (rhomb). Rhombomeres (rh) and rh boundaries (rhb) indicated by dashed lines. **(E?)** Enlargement of the rh region; arrowheads pointing to short primary cilia. **(E?)** Enlargement of the rhb with cells bearing elongated monocilia. **(F, G)** Close-up views onto the fourth ventricle cp showing MCCs. a = anterior; di = diencephalon; hy = hypophysis; l = left; mes = mesencephalon; ml = midline; p = posterior; Po = preoptic region; r = right; tel = telencephalon.

**Figure 4:**
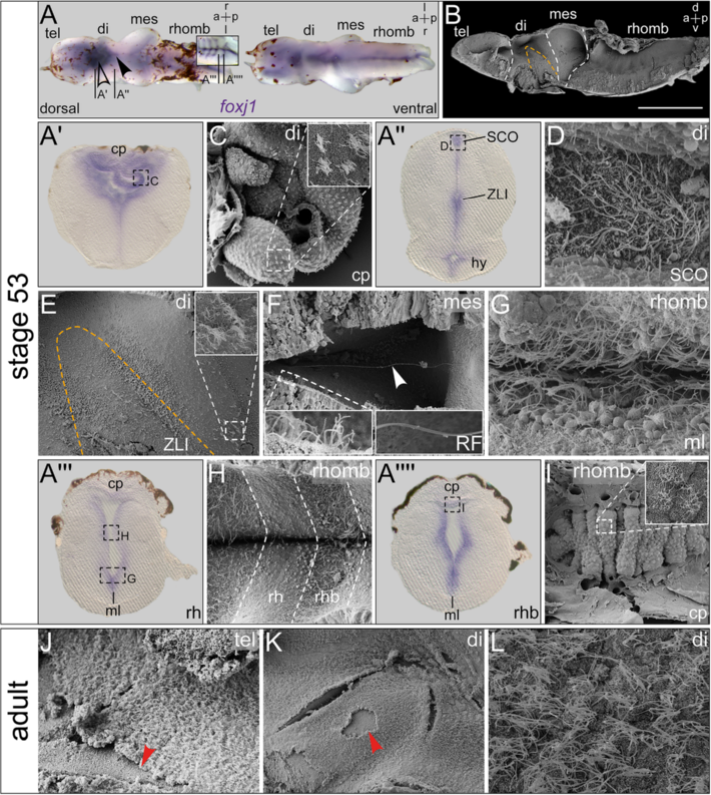
**Continued *foxj1 *expression and ciliogenesis in the premetamorphic and adult brain of *Xenopus. ****In situ* hybridization and scanning electron microscopy (SEM) on explanted brains at stage 53 and SEM on adult brains. **(A)** Explant shown in dorsal (d) and ventral (v) view. Expression in the ventral midline, subcommissural organ (SCO, arrowhead), choroid plexus (cp; outlined arrowhead), and ventricular regions. Inset in **(A)** focuses on expression in rhombomere boundaries (rhb) after removal of the pigmented rhombencephalon (rhomb) roof. **(B)** SEM picture of brain explant dissected sagittally with view onto the ventricular surface, the zona limitans intrathalamica (ZLI) is delimited with orange dashed line and boundaries between brain regions are indicated by white dashed lines. Bar represents 500 ?m. **(A?-A??)** Transversal histological sections as indicated in **(A)**, dorsal side up. **(C-I)** SEM pictures corresponding to regions indicated in **(A?-A??)**, respectively. **(C)** Close-up view of third ventricle cp; inset showing enlarged view of MCCs. **(D)** Top view onto ventricular surface of SCO of a frontally bisected specimen. **(E)** Overview of the diencephalon (di) region; inset showing MCCs emerging at the di-/mesencephalic boundary, similar to those of the ZLI. **(F-I)** Frontally bisected specimens. **(F)** View into the dorsal ventricular lumen of the mesencephalon (mes) with Reissner?s fiber (RF, arrowhead) spanning the ventricle. Left inset: enlargement of mes MCCs. Right inset: enlargement of RF. **(G)** View onto the ventral midline (ml). **(H)** Ventral part of the rhomb with rhombomeres (rh) and rhb as indicated. **(I)** cp of the rhomb roof with MCCs. **(J-L)** SEM pictures of sagittally bisected adult brain in the telencephalon (tel; **(J)**) and di region **(K, L)** showing a multiciliated ependymal monolayer. Arrows indicate sites of ependyma detachment due to mechanical stress during processing. a = anterior; hy = hypophysis; l = left; p = posterior; r = right.

### Continued *foxj1* expression correlates with elongation of monocilia and the emergence of multiciliation

In order to investigate whether sites of *foxj1* expression correlated with cilia formation, whole-mount explants of brains were analyzed in parallel by scanning electron microscopy (SEM). The first stage for which brains could be successfully explanted was stage 35. Earlier specimens frequently ruptured due to the high content of yolk, which was consumed around stage 35. *foxj1* continued to be expressed in the floor plate, ZLI and SCO at that stage (Figure?2A). Brains cut sagittally along the midline and prepared for SEM analysis revealed the internal organization of the ventricular surface; pros-, mes-, and rhombencephalon could be easily distinguished (Figure?2B). The rostral part of the prosencephalon was strongly bent downward and brains measured about 1 mm in length (measured as a straight line from the rostral-most extension of the prosencephalon to the rhombencephalon/spinal cord transition; Figure?2B). A furrow developing in the preoptic region was visible as well, which delineated the developing boundary between tel- and diencephalon as well as the epiphysis (pineal gland) on the dorsal roof of the posterior prosencephalon (Figure?2B). The ZLI was obvious as a V-shaped wedge of tissue in the posterior prosencephalon. The metameric organization of the rhombencephalon was easily discernible by the boundaries between the rhombomeres which appeared as vertical slit-like structures (Figure?2B).

The ependymal surface of stage 35 brains was predominantly constituted by cells which harbored short monocilia (1?2 ?m; Figure?2C, D; arrowheads in Figure?2C?, D?). In addition, cells with single elongated cilia were identified (4?6 ?m; Figure?2C, D; outlined arrowheads in Figure?2C?, D?, E), as well as first oligociliated cells (red arrowhead in Figure?2E). The localization of ciliated cells correlated with preceding expression of *foxj1*, as cells with elongated monocilia appeared in the ZLI (Figure?2C) as well as in the rhombencephalic ventral midline (Figure?2D). Cells of the rhombencephalon roof also harbored elongated monocilia, where several oligociliated cells were intermingled as well (Figure?2E).

At stage 40, *foxj1* expression was found generally unchanged in brains of comparable morphology (Additional file 1: Figure S1A, B). The brain had begun to straighten, a process which continued through stage 43. Brain length at stage 40 still was about the same as at stage 35. Tel-, di-, mes-, and rhombencephalon were easily distinguishable (Additional file 1: Figure S1A, B). While primary cilia in most brain regions remained short (1?2 ?m, Additional file 1: Figure S1C, D; arrowheads in Additional file 1: Figure S1C?, D?), elongated cilia continued to appear and reached maximum lengths of about 9 ?m (Additional file 1: Figure S1C, D; outlined arrowheads in Additional file 1: Figure S1C?, D?). The rhombencephalic roof showed multiciliated cells (MCCs) with cilia of 4?5 ?m in length (arrowhead in Additional file 1: Figure S1E). Around stage 45, the pattern of *foxj1* expression in the brain was supplemented by two additional domains - one spot just anterior to the SCO in the telencephalon (outlined arrowhead in Figure?3A) and a striped pattern in the rhombencephalon (Figure?3A). The completely straightened brain still measured about 1 mm in length and was entirely surrounded by the dura mater. The ventricle underlying the optic tectum widened and invagination of the third ventricle CP began (Figure?3B).

Histological vibratome sections of *in situ*-hybridized brains were used for more detailed correlation of *foxj1* expression and ciliation patterns. Transverse sections through the anterior spot-shaped expression domain of *foxj1* revealed staining in the developing CP of the lateral/third ventricles (Figure?3A?). The CP starts to grow and invaginate into the ventricular system at stage 43 [26]. It became visible as a thickened indentation of the dorsal roof at the tel-/diencephalon junction by SEM, and its ventricular surface was covered densely with MCCs (Figure?3C). In sections at the level of the di-/mesencephalic boundary, staining in both the SCO and ZLI was apparent (Figure?3A?). In the SCO, SEM pictures revealed elongated cilia, to which uncharacterized extracellular material was attached (Figure?3D). The SCO generates Reissner?s fiber (RF), which is composed of aggregated glycoproteins such as SCO-spondin. The RF forms a thread which runs through the entire ventricular system and central canal of the spinal cord [27]. The material sticking to SCO cilia perhaps reflected the production of RF in *Xenopus*, which we frequently witnessed spanning the ventricular lumen (not shown; cf. Figure?4F). The ventricular surface area corresponding to sites of *foxj1* expression found in the ZLI in transverse sections consisted of cells with elongated monocilia, comparable to those seen in the ZLI in stage 35/40 embryos (Figure?3D).

Higher magnification of the ventricular surface of the rhombencephalon in dissected brains (Figure?3A) as well as in transverse sections (Figure?3A??, A??) identified the striped pattern on the lateral walls of the fourth ventricle as the metameric organization of the rhombencephalon. While cells located at the center of the rhombomeres were free of *foxj1* expression (rh; Figure?3A??), transcripts were found in cells at the rhombomere boundaries (rhb; Figure?3A??). This expression pattern was reflected by a differential organization of cilia. Cells with short monocilia covered the *foxj1*-negative regions of the rhombomeres (Figure?3E and arrowheads in E?), whereas the *foxj1*-positive cells at the boundaries of the rhombomeres carried monocilia of 9?10 ?m length (Figure?3E, E?).

Similar to the lateral/third ventricles, the roof of the fourth ventricle starts to thicken and a highly vascularized CP epithelium forms around stage 43 [26]. Cross-sections through the fourth ventricle CP at stage 45 showed strong expression of *foxj1* (Figure?3A??, A??) and SEM revealed the presence of multiciliary tufts on the surface of fourth ventricle CP cells (Figure?3F, G). By stage 53, *foxj1* expression had become very pronounced, extending to all ventricle-lining epithelia (Figure?4A). Brains measured about 2 mm in length and all parts were well developed (Figure?4B). Staining in the medial and lateral domains of the CP at the boundary between the lateral and third ventricle intensified considerably over time, concomitant with the growth and vascularization of the CP tissue, which protruded into the ventricular lumen at stage 53 (Figure?4A?). SEM revealed the highly convoluted arrangement of blood vessels covered with MCCs (Figure?4C).

Sections running through the di-/mesencephalic boundary showed a continually strong expression of *foxj1* in the SCO and ZLI. Additionally, *foxj1* started to be expressed in the ventricular layer of the fully developed pituitary (hypophysis (hy); Figure?4A?). The continuous expression of *foxj1* in the SCO and ZLI was reflected by the morphological development of cilia in these regions. Cilia in the SCO were elongated, however, whether cells of the SCO were mono- or multiciliated could not be discerned (Figure?4D). In the ZLI and at the di-/mesencephalic boundary, the first multiciliary tufts became apparent on the surface of cells (Figure?4E). Corresponding to the onset of *foxj1* expression, luminal cells of the neurohypophyseal infundibulum bore elongated monocilia and in several cases, multiciliary tufts were visible on cells of the infundibular roof (Additional file 2: Figure S2).

In the cerebral aqueduct (CA), cells were studded with elongated monocilia and RF frequently spanned the aqueduct lumen (Figure?4F). Similar to all other ventral regions of the stage 53 brain, the ventral midline of the rhombencephalon was covered with long mono- and multicilia (Figure?4G). The striped expression of *foxj1* in the rhombencephalon (Figure?4A, A??, A??) and the corresponding ciliation pattern was still obvious. While monocilia at the center of rhombomeres remained short, cilia in the boundary regions elongated further and the boundary domain appeared wider (Figure?4H). Similar to the development of the lateral/third ventricle CP, the CP of the rhombencephalon roof developed into an elaborate convoluted structure, the surface of which was covered with MCCs (Figure?4I). In the adult brain, ventricle-contacting cells had developed into a single-layered ependymal epithelium which became apparent in areas, in which the continuous layer had been disrupted during fixation and processing of specimens (arrowheads in Figure?4J, K). Close-up views revealed that the adult ependyma consisted of MCCs throughout (Figure?4L).

Taken together, neural expression of *foxj1* correlated with ciliation patterns in *Xenopus* tadpole brains, such that the first elongated cilia arose in regions of early *foxj1* expression. Sustained transcription in these and additional regions mirrored the continuous development from short to elongated monocilia, eventually resulting in multiciliation of the entire brain ependyma.

### *foxj1* loss of function induces hydrocephalus

To evaluate the function of motile cilia for CSF movement during brain development, we knocked down *foxj1* using a previously characterized antisense morpholino oligonucleotide (MO) which targeted the transcriptional start site [14,28]. *foxj1*MO or a control MO (coMO) were co-injected with a fluorescent lineage tracer into the animal region of dorsal blastomeres of four to eight cell embryos (Figure?5A). Injections were targeted towards the A1 lineage, which has major contributions to brain and spinal cord [29-31]. Correct targeting was assessed when embryos reached neurula stages (Figure?5A). Incubation of injected specimens was continued until stage 46, when they were processed for morphological/SEM or CSF flow analysis (Figure?5A). In all experiments uninjected as well as coMO-injected embryos were used as controls. Morphants were frequently characterized by edema and shortened body axes (not shown). Strikingly, the diencephalic region was massively reduced upon *foxj1* knock-down (Figure?5B, C; Additional file 3: Figure S3) and the brain showed a massive dilatation of the fourth ventricle (Figure?5D, E). This hydrocephalic inflation was clearly visible in whole-mount embryos as well as in explanted brains and cross-sections thereof (Figure?5C, E; Additional file 3: Figure S3).

**Figure 5:**
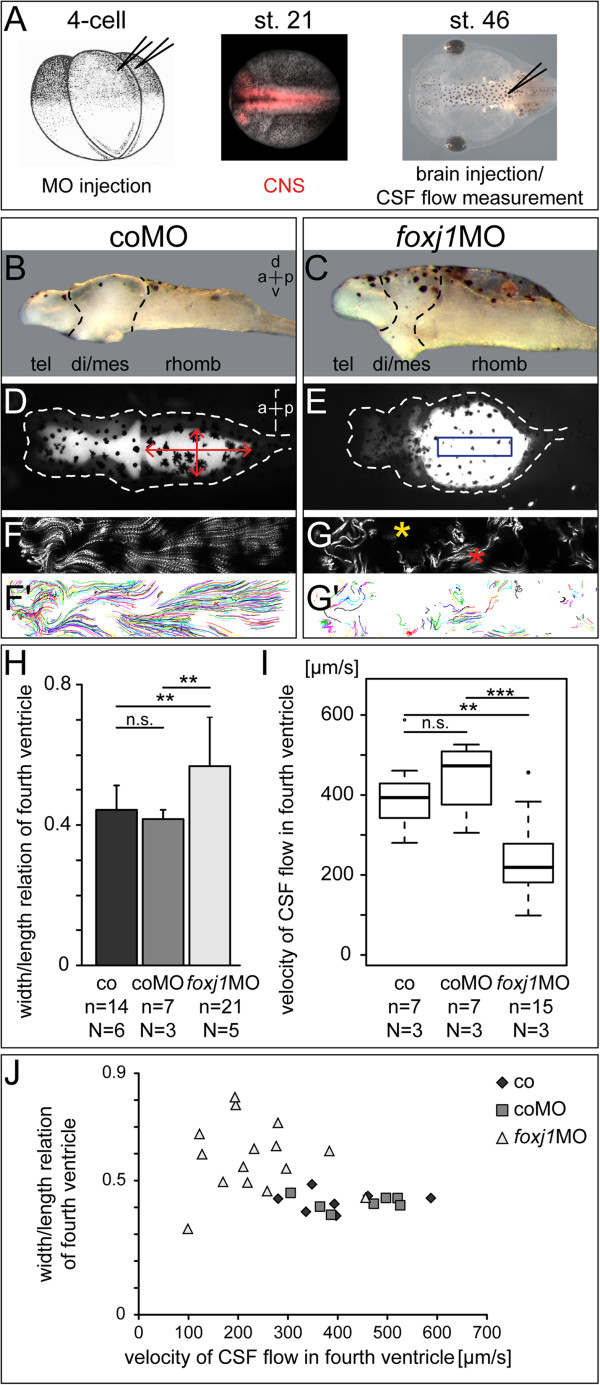
**Loss of function of *foxj1 *in the central nervous system induces hydrocephalus. (A)** Timeline of experimental process. **(B**, **D**, **F**, **F**?**)** Control morpholino (coMO) -injected specimen. **(C**, **E**, **G**, **G**?**) ***foxj1* morpholino (*foxj1*MO) -injected specimen. **(B**, **C)** Brain explants of stage 46 tadpoles, side view. **(D**, **E)** Dorsal (d) view of tadpole heads at stage 46; ventricular system visualized by injection of a fluorescent dye. Note the obstruction of the ventricular system and the hydrocephalic hindbrain ventricle in *foxj*1MO -injected embryos in **(E)**. **(F**, **G)** Z-projection of video-tracked fluorescent beads. **(F**?, **G**?**)** Display of statistically analyzed bead trajectories. **(H)** Statistical analysis of fourth ventricle width to length relation, measured as indicated in **(D)**. 0 = linear ventricle, 1 = perfect circle; error bars represent SD. **(I)** Statistical analysis of CSF flow velocity in the fourth ventricle. Region of interest chosen for flow measurement as indicated by blue box in **(E)**. **(J)** Correlation of statistical analyses in **(H)** and **(I)**. a = anterior; CNS = central nervous system; co = control; CSF = cerebrospinal fluid; di = diencephalon; l = left; mes = mesencephalon; n = number of specimens; N = number of experiments; p = posterior; r = right, rhomb = rhombencephalon; tel = telencephalon; v = ventral.

CSF flow analysis following injection of both dye solution and microbeads revealed even distribution throughout the ventricular system of control embryos (Figure?5D). However, fluorescent signals were frequently absent from the forebrain of *foxj1* morphant specimens (Figure?5E). In the few cases when dye penetrated into the forebrain, ventricles appeared severely misshapen (Additional file 3: Figure S3). Hindbrain ventricles, however, were always completely filled with dye and markedly inflated in *foxj1* morphants (Figure?5E; Additional file 3: Figure S3). Hydrocephalic expansion of the fourth ventricle was quantitatively assessed by calculating the width-to-length ratio in control and *foxj1*MO-injected embryos (red arrows in Figure?5D). Compared to either control type, in which the fourth ventricle appeared elliptical, the ventricle shape of *foxj1* morphants was shifted towards a more circular appearance (Figure?5H; *P* = 0.00191 and *P* = 0.001541).

In-depth flow analysis by time-lapse videography revealed connectivity between all ventricles as well as CSF circulation throughout in control specimens. Beads were propelled rostrally in the dorsal regions of the ventricles, while in ventral domains, beads were moved caudally (not shown), as previously described [32]. CSF/bead movement was most easily observed at the CP epithelia of the lateral/third and fourth ventricles. Since forebrain ventricles were often collapsed in *foxj1* morphants, flow was monitored at the fourth ventricle CP, that is, the dorsal roof of the rhombencephalon. The dominant pattern of bead movement in the fourth ventricle was a fast and laminar rostral flow close to the dorsal ventricular surface (Figure?5F, F?; Additional file 4: Movie S5). Beads were propelled downward in proximity to the rostral end of the ventricle and converged into a slower caudal-ward flow along the ventral midline. In the medial regions of the ventricle, beads moved laterally and dorsally to transition back into the fast rostral-ward stream along the rhombencephalon roof (Additional file 4: Movie S5).

In order to image cilia-driven flow without the influence of the dorsal MCCs of the rhombencephalon roof, brains were excised and dissected along the dorsal midline to remove the dorsal roof. Explants were flat-mounted in fluid-filled chambers, which contained beads to image flow on the ventral and lateral ventricular surface of the fourth ventricle. Fluid flow driven by motile cilia was detected both medially along the ventral midline as well as lateral to the midline. Lateral flow was linear from rostral to caudal, however, it was interrupted and re-initiated by the motile cilia at the rhombomere boundaries (Additional file 5: Movie S6). Analyses of CSF flow thus strongly suggested that all elongated cilia in the rhombencephalon, including the monocilia at the rhombomere boundaries, were motile and produced fluid flow. The data also demonstrated that multiciliated CP cells were the driving force of CSF flow, which are necessary and sufficient to generate the circulatory movement of fluid detected in the fourth ventricle.

For a detailed analysis of CSF flow parameters, regions of interest were chosen at the center of the ventricle (blue box in Figure?5E) and the focus plane was adjusted such that laminar flow driven by ciliary bundles on the dorsal roof was recorded (refer to Figure?3F, G). Beads were rapidly propelled in a caudal to rostral fashion; bead movement was visualized by maximum z-projection of the time series and trajectories computed by the ImageJ plug-in ParticleTracker (Figure?5F?, G?; Additional file 6: Movie S7). Comparison of trajectories from control and *foxj1* morphant specimens revealed three prominent features: (1) CSF flow was severely impaired in morphants; (2) the degree with which cilia were lost on cells of the rhombencephalon roof varied. In some patches beads were still propelled in a directed manner (red asterisk in Figure?5G), whereas no moving beads were detected in between these patches (yellow asterisk in Figure?5G), indicating that in such areas cells had lost motile cilia altogether; (3) bead velocities were compromised in morphants (Figure?5I). In uninjected control embryos, the mean velocities were determined at 401 ? 100 ?m/s. coMO-injected specimens ranged at around an average of 439 ? 86 ?m/s, a deviation that was not statistically significant (*P* = 0.4557). *foxj1* morphants, in contrast, showed a significant reduction of bead velocities with 235 ? 96 ?m/s (*P* = 0.0011 morphant *vs*. uninjected; *P* = 0.00022 morphant *vs*. coMO). When ventricle shape (Figure?5H) of individual embryos was plotted against flow velocity (refer to Figure?5I), the shape of ventricles was consistently elliptical within the control group (Figure?5J), even though flow velocities in control specimens varied between 300?600 ?m/s. In *foxj1* morphants, CSF flow velocity dropped to <300 ?m/s which strongly correlated with a bias towards a rounded appearance of the ventricle, that is, hydrocephalic inflation. In summary, loss of *foxj1* in the *Xenopus* CNS resulted in hydrocephalus of the fourth ventricle and a significant decrease in CSF flow velocity.

### CSF flow velocity is caused by motile cilia

To confirm that reduction of CSF flow velocity was caused by a loss of motile cilia, ciliation in *foxj1*MO- and coMO-injected brains was assessed by SEM. Brains of coMO-injected specimens appeared morphologically normal with a total length of about 1 mm and readily distinguishable brain regions (Figure?6A). As in wild-type brains, elongated cilia were visible in the ZLI and SCO (Figure?6B, C), and the rhombencephalon showed the conspicuous banded pattern (short monocilia at the center and elongated cilia at the rhombomere boundaries; Figure?6D). The rhombencephalon roof was covered with MCCs throughout (Figure?6E). *foxj1* morphant brains were noticeably shorter, measuring about 0.6 mm in length. This shortening was due to a reduction of the di-/mesencephalic area, in which the boundaries between di- and mesencephalon had become almost indistinguishable. The anterior-posterior extension of the rhombencephalon remained unchanged, while the dorso-ventral dimension (height), measured as the distance between ventral midline and roof, increased about two-fold (Figure?6F; refer to Figure?5E, H). While wild-type and coMO brains harbored elongated monocilia on ventricle-contacting ZLI cells at stage 46, most ZLI cells in *foxj1* morphant brains lacked elongated cilia and displayed only short monocilia (Figure?6G). The surface of the SCO was markedly altered in *foxj1* morphant embryos. Individual cilia were not detected and the surface of the SCO was covered by extracellular material appearing like a cobweb (Figure?6H). Knock-down of *foxj1* had dramatic effects on ciliation of the rhombencephalon as well. While short and stubby monocilia persisted, cells at the rhombomere boundary lost their long monocilia, displaying a microvillous coat instead (Figure?6I). MCCs on the CP of the rhombencephalon roof were likewise affected. In most cases, only few cells with multiciliary tufts were dispersed across the ventricular surface of the CP (arrowheads in Figure?6J), corresponding to the few scattered areas of fluid flow seen in brains of *foxj1* morphants (refer to Figure?5G).

**Figure 6:**
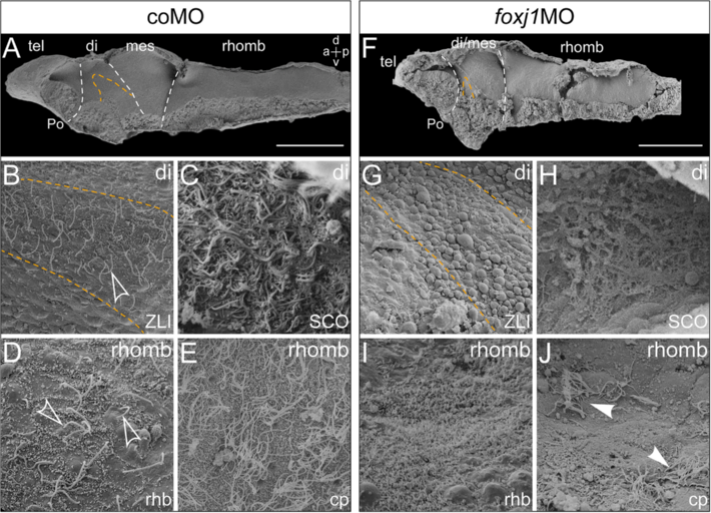
**Reduced CSF flow velocity correlates with defects in ciliogenesis.** Scanning electron microscopy (SEM) of dissected brain explants at stage 46. **(A-E)** Control morpholino (coMO) -injected specimen. **(F**-**J)***foxj1* morpholino (*foxj1*MO) -injected specimen. **(A**,**F)** SEM pictures of brain explants dissected sagittally with view on the ventricular surface, the zona limitans intrathalamica (ZLI) is delimited with orange dashed line and boundaries between brain regions indicated by white dashed lines. Bars represent 200 ?m. **(B**,**G)** View onto the ZLI in the diencephalon (di). Note the loss of cilia on the ZLI in *foxj1* morphants **(G)**. **(C**,**H)** Close-up views onto the subcommissural organ (SCO) in frontally dissected specimens. **(D**,**I)** Close-up views onto the rhombencephalon in sagittally dissected specimens, depicting the rhombomere boundaries. Outlined arrows in **(D)** point to elongated monocilia. Note the absence of cilia in **(I)**. **(E**,**J)** Close-up view onto the choroid plexus (cp) of the hindbrain roof. Arrowheads in **(J)** point to the few remaining MCCs in *foxj1* morphants. a = anterior; d = dorsal; mes = mesencephalon; tel = telencephalon; rhomb = rhombencephalon; Po = preoptic region; p = posterior; rhb = rhombomere boundary; v = ventral.

In summary, our analyses revealed a critical role of *foxj1* for motile cilia and CSF flow in the developing *Xenopus* brain. Our data underscore the function of CSF flow for brain morphogenesis and establish a connection between impaired ciliary motility and hydrocephalus.

## Discussion

### Expression of *foxj1* precedes and is spatially correlated with the emergence of elongated cilia

During vertebrate embryonic development, all neuroepithelial and radial glia cells initially carry an immotile monocilium on their apical surface which faces the ventricular lumen [33]. In the mouse, differentiation of the ventricle-lining ependyma into a single layer of cuboidal MCCs occurs towards the end of the first postnatal week [34]. Neural expression of *Foxj1* precedes the emergence of ependymal cilia, as transcripts were detected during mouse embryonic development. Sites of *Foxj1* transcription during mouse neurulation (embryonic day E10/13.5) include the rostral-most rhombomeres, mid-/hindbrain boundary, caudal midline, mesencephalic vesicle, ventro-lateral forebrain and CPs of the lateral, third, and fourth ventricles [12,35]. *Foxj1* is thus expressed in homologous regions during embryogenesis of mouse and frog. If one considers the onset of metamorphosis in amphibians roughly equivalent to birth in mammals, also the time course of *Foxj1* expression in the mouse brain complies with our observations in the *Xenopus* brain. Preceding the general appearance of ependymal cilia in the mouse, MCCs have been identified in the epithelia of the mesencephalic cerebral aqueduct as well as the CP at postnatal day (P) 1 [36]. Beyond CP and aqueduct we identified additional sites of ciliation in the *Xenopus* tadpole brain, in particular in the SCO, ZLI, and ventro-lateral rhombencephalon, all of which expressed *foxj1* prior to the elongation of cilia. It will be interesting to analyze whether the *foxj1*/cilia module at these sites is conserved in the mouse as well.

Transcription factors of the RFX family governing ciliogenesis are also expressed in neural tissue during *Xenopus* embryonic development [37]. However, while RFX family members 1?5 are found throughout the sensorial layer during neural fold apposition and closure, *foxj1* is restricted to the ventral midline and floor plate at the same stages. This illustrates that RFX factors and *foxj1* have overlapping yet distinct functions in ciliogenesis. While RFX factors in neural tissue seem to be required for both the primary cilia of sensorial layer cells and the prospective motile cilia of the floor plate, *foxj1* acts as the master regulator for motile cilia.

Interestingly, during late embryogenesis (stage 45/46), when vigorous and rapid CSF flow has developed, only a few areas of the brain expressing *foxj1* were multiciliated, while the remainder of *foxj1*-expressing cells harbored elongated monocilia. Multiciliation developed later in areas with sustained *foxj1* expression. This observation is in perfect agreement with the notion that low levels of *foxj1* expression induce cells to form an elongated motile monocilium [14]. The switch of a cell?s ciliation from an elongated motile monocilium to multiple motile cilia requires expression of the small coiled-coil domain-containing protein multicilin [28]. Based on the time points of MCC emergence identified here, it will be interesting to analyze the appearance of multicilin expression in the *Xenopus* brain.

### CSF flow is driven by motile cilia

Two recent studies have described CSF flow patterns in the *Xenopus* tadpole brain [32,38]. Motile cilia were proposed as a possible cause, based on reports of ciliated ventricles in other anuran species [39,40]. Heartbeat was discussed as an alternative or additional mode of CSF flow control, as anesthesia of tadpoles with MS-222, which resulted in slowed or absent heartbeat, reduced flow velocities from 77 ?m/s to 11 ?m/s [38]. MS-222 is known to acidify aqueous solutions, and low pH in turn reduces ciliary beat frequency (CBF) [41]. We used benzocaine as an anesthetic and our experimental setup was designed to limit dose (<0.01%) and incubation time prior to bead injection (<5?) as much as possible, which might explain why we have not encountered heartbeat-dependence of CSF flow. The overall much higher CSF flow velocities in our study (450 ?m/s) might be explained through avoidance of unspecific effects on pH and CBF as well. Taken together our data strongly suggest that cilia are the sole source of CSF flow in *Xenopus*, notwithstanding the role of blood flow in maintaining the physiological function of the CP.

### Absence of motile cilia causes hydrocephalus

Even though mutations in cilia-associated genes are well-documented in the etiology of hydrocephalus [42,43], the mechanism of how a loss of motile cilia causes hydrocephalic inflation of the ventricles has remained unclear. Since the loss of motile cilia in *foxj1* morphants affects diverse cell populations in the developing brain it is conceivable that the development of hydrocephalus might be multifactorial.

In our *foxj1* gene knock-down experiments, cilia in the *Xenopus* ventricles were not entirely absent. Consequently, flow was never completely abolished, although velocities were significantly reduced. Remarkably, a drop below a critical threshold of 300 ?m/s was correlated with fourth ventricle hydrocephalus in individual morphant tadpoles. The rostrally directed CSF flow at the roof of the rhombencephalon presents the most vigorous one of the entire ventricular system (450 ?m/s *vs*. 160 ?m/s at the anterior CP). Flow rates <300 ?m/s might fail to create enough pressure to force CSF through the aqueduct into the forebrain ventricles. It is tempting to speculate that this failure of CSF penetration into the forebrain could result in a secondary effect - a collapse of forebrain ventricles, which we have frequently witnessed in *foxj1* morphant brains (Additional file 3: Figure S3).

A collapse of the forebrain ventricles or stenosis of the mesencephalic aqueduct might alternatively result from *foxj1* loss of function in the fore-/midbrain area. Aqueduct stenosis preceding the occurrence of hydrocephalus has been described in rats in which the formation of RF by the SCO was manipulated [44]. This notion was recently supported by an analysis of rats with morphological and functional deficiency of the SCO [45]. RF is thought to keep the aqueduct open, and manipulations interfering with secretion or aggregation of RF-glycoproteins lead to collapse of the aqueduct and subsequent hydrocephalus in late postnatal stages. In *Xenopus*, Reissner?s substance (the RF precursor) is first produced by cells of the floor plate and subsequently by the developing SCO [46]. Both floor plate and SCO are regions with prominent *foxj1* expression and ciliation in the SCO has been severely affected in our *foxj1* knock-down experiments (Figure?6). The loss of motile cilia through depletion of *foxj1* might affect the production and/or distribution of RF, causing aberrant ventricular morphology in the fore- and midbrain and in the end hydrocephalus of the hindbrain. Aqueduct stenosis has also been described as the cause of hydrocephalus occurring postnatally in mice deficient for *Mdnah5*, which show loss of ciliary motility on ependymal cells [5].

However, hydrocephalus can also occur independently of aqueduct stenosis as seen in RFX3-deficient mice [8]. Here, loss of RFX3 causes aberrant ciliation in the SCO and a decrease in RF immunoreactivity, leading to congenital hydrocephalus without collapse of the aqueduct. Even though SCO ciliation is altered in RFX3 mice, ependymal cilia are motile. It thus seems plausible that the correct inflation and shape of the ventricular system is determined by an interplay of ciliary motility, developmental RF production, and postnatal SCO function. Variations in these factors might determine some of the morphological differences. Future analyses will evaluate to what degree loss of function of *foxj1* causes perturbations in the SCO and impairment of RF production.

Another possible origin of hydrocephalus might be an overproduction of CSF, leading to increased fluid pressure in the ventricular system. In the mouse, dysfunctional cilia on the CP have been implicated in the misregulation of CSF production [36]. As the entire roof of the *Xenopus* rhombencephalon constitutes a CSF-secreting CP, which was impaired in *foxj1* morphants, it is conceivable that an altered secretory activity of CP cells may have led to an overproduction of CSF as well.

Remarkably, *foxj1* knock-down led to conspicuous morphogenetic changes in the di-/mesencephalon as well, reflected by a shortening of the affected area along the rostro-caudal axis. The affected area harbors the ZLI, an important signaling center for thalamus determination during embryonic brain development. Other signaling centers in the embryonic CNS comprise the floor plate, the isthmus (mid/hindbrain) organizer, and the rhombomere boundaries. Interestingly, rhombomere boundary cells expressed *foxj1* and had a single elongated motile cilium on their surface. Although the functional significance of this correlation remains elusive, it is well established that rhombomere boundary cells serve as signaling centers, involved in the maintenance of the metameric identity of the hindbrain [47-49]. A defining characteristic of all the aforementioned signaling centers seems to be that they express *foxj1* and develop single elongated motile cilia (Additional file 7: Figure S4). These cilia may play a pivotal role for the functionality of these signaling centers, through propagation of morphogen gradients and/or detecting and processing signaling molecules. The patterning of the rhombencephalon is governed by three signaling centers (floor plate, rhombomere boundaries, and isthmus organizer). The loss of motile cilia in each of these centers in *foxj1* morphants may thus disturb hindbrain morphogen gradients, and together with reduced flow velocity and aqueduct stenosis cause hydrocephalic enlargement of the fourth ventricle. Taken together, the absence of motile cilia may impact on a multitude of processes in diverse parts of the brain, some or a combination of which may result in the development of hydrocephalus.

## Conclusions

Hydrocephalus presents a common birth defect in humans, occurring with a frequency of 0.1-0.3% of live births [50]. Among other causes, congenital hydrocephalus has been attributed to a loss of ciliary motility in the brain ventricles and the consequential loss of CSF movement. The *Xenopus* tadpole brain constitutes an attractive novel model system to study the formation of hydrocephalus during embryogenesis. The present study provides a reference point with its precise account of the temporal-spatial appearance of motile mono- and multiciliated cells and the assessment of CSF flow in live specimens. Morphant tadpoles deficient in *foxj1*, which lack motile cilia and consequently develop hydrocephalus, demonstrate the general validity of the system. Emerging new techniques for gene knock-down and allele replacement in *Xenopus* should offer an opportunity to directly analyze the role of human genes whose deficiency may cause hydrocephalus formation.

## Competing interests

The authors declare that they have no competing interests.

## Authors? contributions

CH co-designed the study, carried out the majority of the experiments, analyzed and interpreted the data, prepared the figures, and helped to draft the manuscript. PW contributed to the study design, to the *foxj1* loss of function experiments, and prepared *in situ* hybridizations. CM contributed *in situ* hybridizations. TT developed the semi-automated flow analysis tool, adapted it for this study, and helped substantially with analysis and presentation of CSF flow data. KF conceived, designed and supervised the study, interpreted the data, and wrote the manuscript. All authors read and approved the final manuscript.

## Supplementary Material

Additional file 1: Figure S1 *foxj1* expression correlates with elongation of monocilia and the emergence of multiple cilia. *In situ* hybridization and scanning electron microscopy (SEM) on explanted brains at stage 40. **(A)** Explant shown in dorsal (d) and ventral (v) view. Strong expression in the ventral midline and the subcommissural organ (SCO; arrowhead). **(B)** SEM picture of brain explant dissected sagittally with view onto the ventricular surface, the zona limitans intrathalamica (ZLI) is delimited with orange dashed line and boundaries between brain regions indicated by white dashed lines. Bar represents 200 ?m. **(C)** Overview and enlargements show short primary cilia on cells in the diencephalon (di; arrowhead in **(C?)**), and elongated monocilia on cells within the ZLI (outlined arrowhead in **(C?)**). **(D)** Close-up view onto the ventral aspect of a single hindbrain rhombomere with indicated boundaries and midline (ml). Enlargements showing short cilia on the rhombomere (arrowhead in **(D?)**) as well as elongated cilia in the ventral ml (outlined arrowhead in **(D?)**). **(E) **Close-up view onto the rhombencephalon (rhomb) roof with MCCs (arrowhead). a = anterior; l = left; mes = mesencephalon; p = posterior; Po = preoptic region; pros = prosencephalon; r = right; tel = telencephalon. Click here for file

Additional file 2: Figure S2 Expression of *foxj1* in the infundibular wall correlates with emergence of MCCs. *In situ* hybridization and scanning electron microscopy (SEM) on explanted brains at stage 53. **(A)** Right and left hemisphere of brain sectioned sagittally along the midline. The infundibulum is framed by a dashed line. **(B)** Transversal section, as indicated in **(A)** reveals expression of *foxj1* in the zona limitans intrathalamica (ZLI) and the neurohypophysis (nHy) but not in the adenohypophysis (aHy). **(C)** SEM picture of brain dissected sagittally. **(C?)** Close-up view onto the infundibular wall with elongated cilia (arrowheads) and one MCC (outlined arrowhead). d = dorsal; di = diencephalon; mes = mesencephalon; rhomb = rhombencephalon; tel = telencephalon; v = ventral. Click here for file

Additional file 3: Figure S3 Loss of function of *foxj1* shortens the forebrain. **(A)** Statistical analysis of forebrain ventricle length and di- /mesencephalic ventricle width as indicated by colored arrows. **(B-D)** Dorsal view of coMO **(B)** and *foxj1*MO **(C, D)** -injected specimens. **(B?-D??)** Transversal sections as indicated in **(B-D)** with ventricular lumen highlighted in yellow. a = anterior; co = control; di = diencephalon; l = left; mes = mesencephalon; n = number of specimens; N = number of experiments; p = posterior; r = right, rhomb = rhombencephalon. Click here for file

Additional file 4 **Movie S5.** CSF flow in the fourth ventricle. Movie illustrating patterns of cerebrospinal fluid flow in the fourth ventricle of an untreated control embryo at stage 46 upon injection of fluorescent microbeads. Focus plane of the movie shifts in five steps from dorsal (d) to ventral (v) as indicated by the red line in the schematic cross section through the fourth ventricle presented in the inset. Every frame of the movie comprises 20 consecutive raw frames that were color-coded from red to green and projected onto a single frame by using a custom interpretation of the "Temporal-Color Coder" (Kota Miura, EMBL, Heidelberg) plug-in for Fiji. Note the fast rostrally-directed flow just underneath the dorsal roof, change of flow direction in medial focus planes, and caudally-directed flow in the ventral-most focus planes. Movie plays in real time. a = anterior; p = posterior. Click here for file

Additional file 5 **Movie S6.** CSF flow in fourth ventricle explants demonstrates motility of rhombomere and midline cilia. Movie showing caudally directed flow at the ventral midline (medial region of movie) and rhombomere regions (lateral regions of movie) of the fourth ventricle. Explanted brains were opened along the dorsal midline, the dorsal roof was removed and the brain was mounted flat in bead-containing solution (refer to the Methods section). Movie starts with a brightfield image of the region, followed by movement of fluorescent beads and ending in a z-projection of all recorded frames. An interruption and re-initiation of the slow lateral flow by motile cilia at the rhombomere boundaries (indicated by white lines) is visible in the movie and best seen in the final z-projection. Movie plays in real time. a = anterior, p = posterior. Click here for file

Additional file 6 **Movie S7.** Loss of function of *foxj1* decreases fluid flow velocity in the fourth ventricle. Movie showing a comparison of flow at the dorsal roof of the fourth ventricle in uninjected controls (co), control morpholino-(coMO), and *foxj1* morpholino (*foxj1*MO) -injected embryos. coMO and *foxj1*MO embryos are the exact ones depicted in Figure?5E-G and F?, G?. Movie starts and ends with a maximum z-projection of all frames. Every frame of the movie comprises 20 consecutive raw frames that were color-coded from red to green and projected onto a single frame by using a custom interpretation of the "Temporal-Color Coder" (Kota Miura, EMBL, Heidelberg) plug-in for Fiji. Note the rapid anterior-ward movement of beads in co and coMO specimens as compared with slow and aberrant movement of beads in *foxj1*MO embryo. Movie is first shown in real time and then repeated at one fifth of the original speed. a = anterior; p = posterior. Click here for file

Additional file 7: Figure S4 Signaling centers in the central nervous system show elongated monocilia. Scanning electron microscopy pictures of sagittally bisected brain explants at stage 45. **(A)** The isthmus organizer (mid-hindbrain boundary) region; note the presence of several elongated monocilia on the mesencephalic aqueduct side (outlined arrowheads) as well as a population of monociliated cells on the ventral (v) aspect of the isthmus (arrowheads). **(B)** Close-up view onto the lumen of the spinal cord. Arrowheads point to elongated monocilia projecting into the central canal, the appearance of which correlates with expression of *foxj1* (cf Figure?1B-F, Figure?2A). a = anterior; d = dorsal; p = posterior. Click here for file
