# Supplementary figures and images for: Ciliogenesis and cerebrospinal fluid flow in the developing Xenopus brain are regulated by foxj1
Source: Cilia. 2013 Sep 24;2:12. doi: 10.1186/2046-2530-2-12 (PMC3848805; doi:10.1186/2046-2530-2-12)

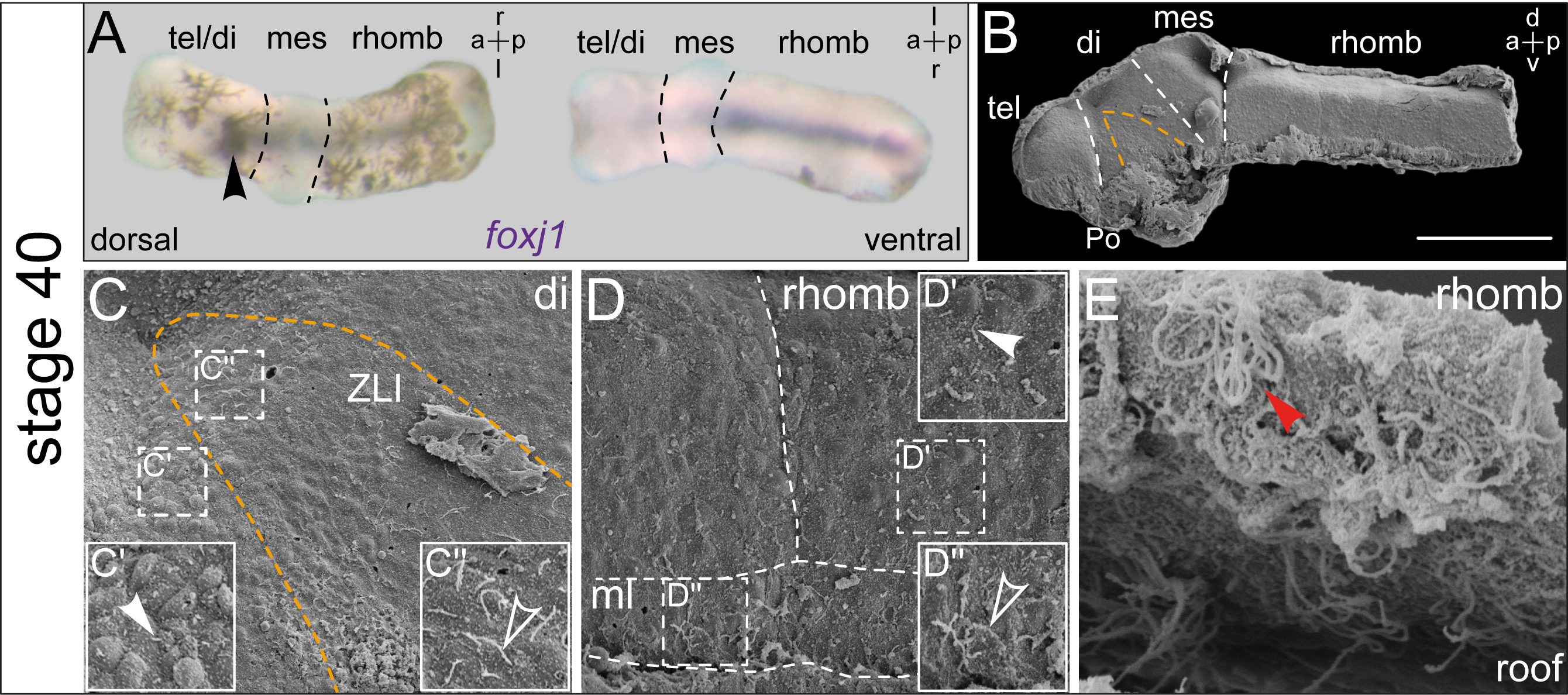

Supplement: Additional file 1: Figure S1 — foxj1 expression correlates with elongation of monocilia and the emergence of multiple cilia. In situ hybridization and scanning electron microscopy (SEM) on explanted brains at stage 40. (A) Explant shown in dorsal (d) and ventral (v) view. Strong expression in the ventral midline and the subcommissural organ (SCO; arrowhead). (B) SEM picture of brain explant dissected sagittally with view onto the ventricular surface, the zona limitans intrathalamica (ZLI) is delimited with orange dashed line and boundaries between brain regions indicated by white dashed lines. Bar represents 200 ?m. (C) Overview and enlargements show short primary cilia on cells in the diencephalon (di; arrowhead in (C?)), and elongated monocilia on cells within the ZLI (outlined arrowhead in (C?)). (D) Close-up view onto the ventral aspect of a single hindbrain rhombomere with indicated boundaries and midline (ml). Enlargements showing short cilia on the rhombomere (arrowhead in (D?)) as well as elongated cilia in the ventral ml (outlined arrowhead in (D?)). (E) Close-up view onto the rhombencephalon (rhomb) roof with MCCs (arrowhead). a = anterior; l = left; mes = mesencephalon; p = posterior; Po = preoptic region; pros = prosencephalon; r = right; tel = telencephalon. [file 2046-2530-2-12-S1.tiff]

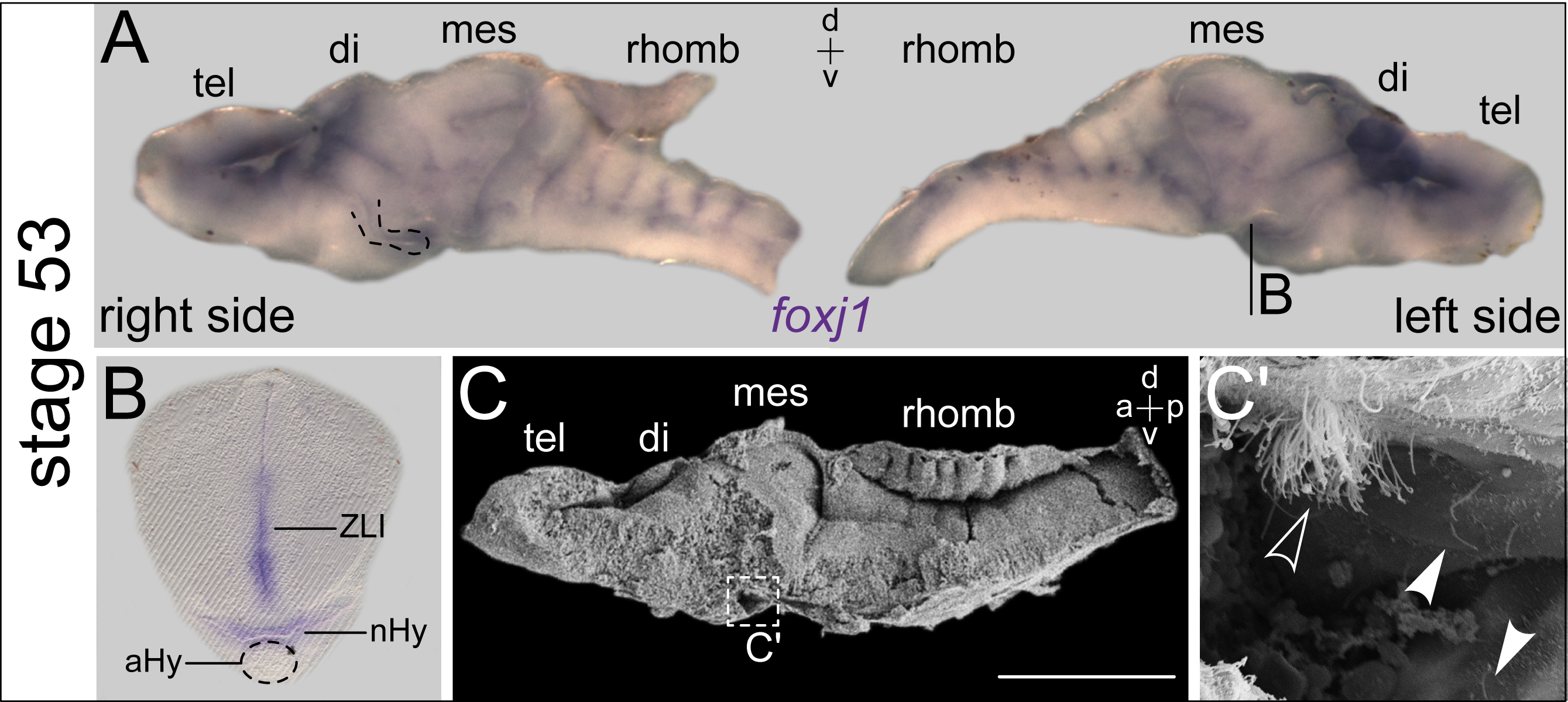

Supplement: Additional file 2: Figure S2 — Expression of foxj1 in the infundibular wall correlates with emergence of MCCs. In situ hybridization and scanning electron microscopy (SEM) on explanted brains at stage 53. (A) Right and left hemisphere of brain sectioned sagittally along the midline. The infundibulum is framed by a dashed line. (B) Transversal section, as indicated in (A) reveals expression of foxj1 in the zona limitans intrathalamica (ZLI) and the neurohypophysis (nHy) but not in the adenohypophysis (aHy). (C) SEM picture of brain dissected sagittally. (C?) Close-up view onto the infundibular wall with elongated cilia (arrowheads) and one MCC (outlined arrowhead). d = dorsal; di = diencephalon; mes = mesencephalon; rhomb = rhombencephalon; tel = telencephalon; v = ventral. [file 2046-2530-2-12-S2.tiff]

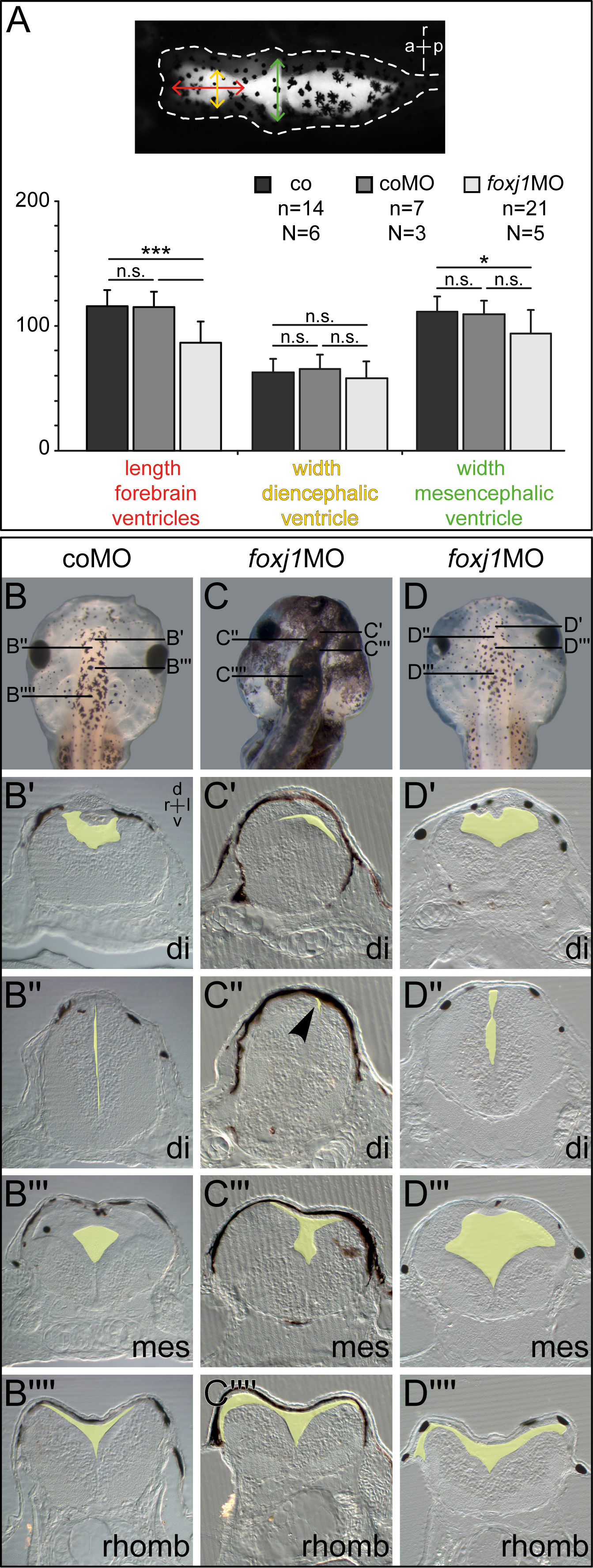

Supplement: Additional file 3: Figure S3 — Loss of function of foxj1 shortens the forebrain. (A) Statistical analysis of forebrain ventricle length and di- /mesencephalic ventricle width as indicated by colored arrows. (B-D) Dorsal view of coMO (B) and foxj1MO (C, D) -injected specimens. (B?-D??) Transversal sections as indicated in (B-D) with ventricular lumen highlighted in yellow. a = anterior; co = control; di = diencephalon; l = left; mes = mesencephalon; n = number of specimens; N = number of experiments; p = posterior; r = right, rhomb = rhombencephalon. [file 2046-2530-2-12-S3.tiff]

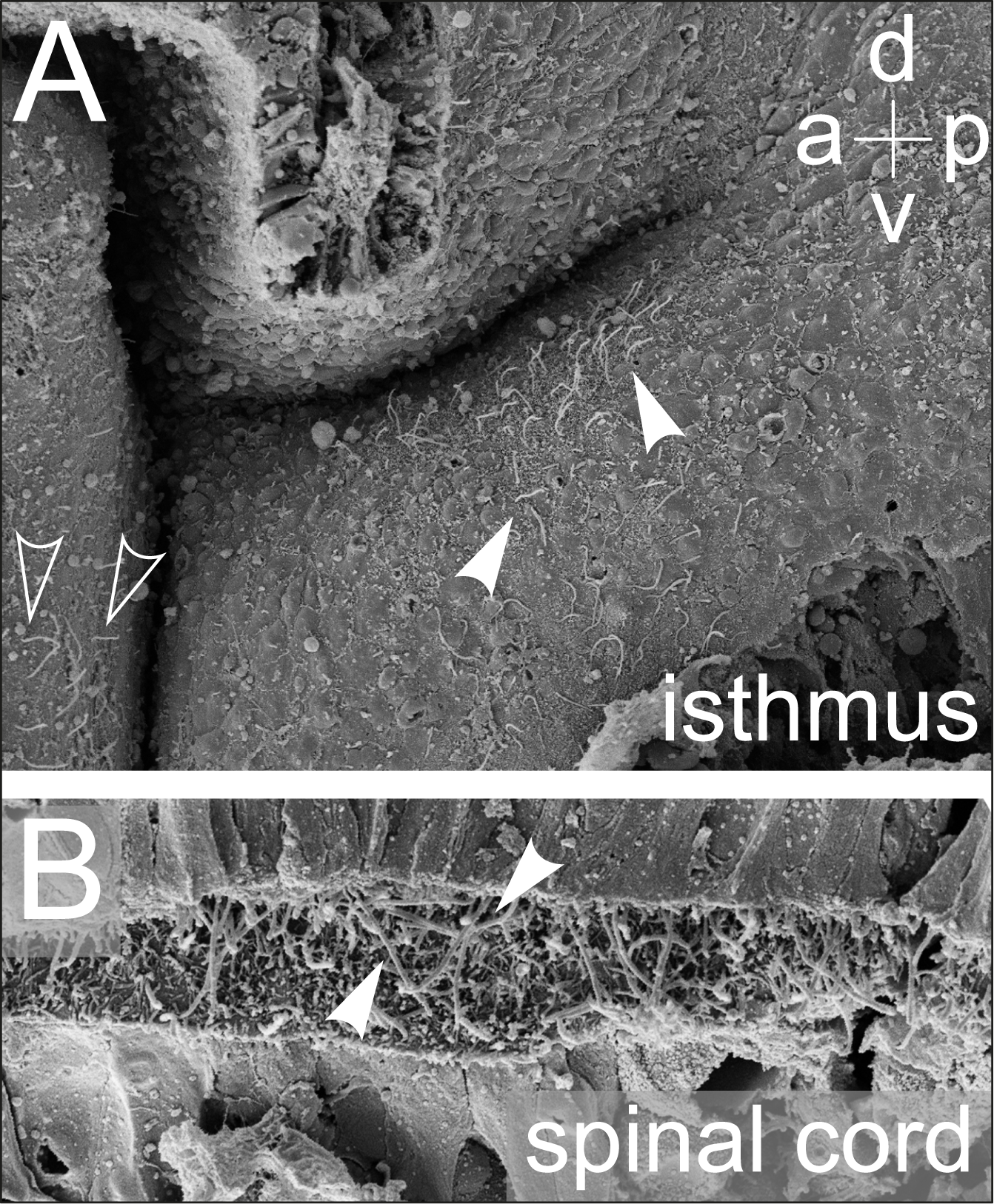

Supplement: Additional file 7: Figure S4 — Signaling centers in the central nervous system show elongated monocilia. Scanning electron microscopy pictures of sagittally bisected brain explants at stage 45. (A) The isthmus organizer (mid-hindbrain boundary) region; note the presence of several elongated monocilia on the mesencephalic aqueduct side (outlined arrowheads) as well as a population of monociliated cells on the ventral (v) aspect of the isthmus (arrowheads). (B) Close-up view onto the lumen of the spinal cord. Arrowheads point to elongated monocilia projecting into the central canal, the appearance of which correlates with expression of foxj1 (cf Figure?1B-F, Figure?2A). a = anterior; d = dorsal; p = posterior. [file 2046-2530-2-12-S7.tiff]
